# Wearable Inertial Measurement Unit Sensing System for Musculoskeletal Disorders Prevention in Construction

**DOI:** 10.3390/s21041324

**Published:** 2021-02-13

**Authors:** Junqi Zhao, Esther Obonyo, Sven G. Bilén

**Affiliations:** 1 Department of Architectural Engineering, The Pennsylvania State University, State College, PA 16802, USA; jzz30@psu.edu; 2 School of Engineering Design, Technology and Professional Programs, The Pennsylvania State University, State College, PA 16802, USA; sbilen@psu.edu

**Keywords:** wearable sensors, IMU, injury prevention, ergonomics, construction safety

## Abstract

Construction workers executing manual-intensive tasks are susceptible to musculoskeletal disorders (MSDs) due to overexposure to awkward postures. Automated posture recognition and assessment based on wearable sensor output can help reduce MSDs risks through early risk-factor detection. However, extant studies mainly focus on optimizing recognition models. There is a lack of studies exploring the design of a wearable sensing system that assesses the MSDs risks based on detected postures and then provides feedback for injury prevention. This study aims at investigating the design of an effective wearable MSDs prevention system. This study first proposes the design of a wearable inertial measurement unit (IMU) sensing system, then develops the prototype for end-user evaluation. Construction workers and managers evaluated a proposed system by interacting with wearable sensors and user interfaces (UIs), followed by an evaluation survey. The results suggest that wearable sensing is a promising approach for collecting motion data with low discomfort; posture-based MSDs risk assessment has a high potential in improving workers’ safety awareness; and mobile- and cloud-based UIs can deliver the risk assessment information to end-users with ease. This research contributes to the design, development, and validation of wearable sensing-based injury prevention systems, which may be adapted to other labor-intensive occupations.

## 1. Introduction

The research presented in this paper is part of a larger project directed at developing a data-driven approach for mitigating the risk of musculoskeletal disorders (MSDs) among construction workers, such as chronic backache and over-exertion. Labor-intensive construction tasks tend to expose workers to using awkward working postures, such as working overhead, kneeling, and back bending. Overexposure to awkward postures can lead to MSDs. Construction-related MSDs account for 30% of workplace injuries in the U.S. [1]. Employers paid around US $53 billion annually on direct cost for MSDs treatment [2] in 2012 to 2014. Proactive MSDs prevention is predicated on effective monitoring of workers’ physical status. However, conventional observation-based risk-monitoring strategies are impractical on construction sites. Safety inspectors can be overwhelmed by jobsite complexity, such as rapidly changing working conditions and concurrent appearance of various construction tasks [3]. Hence, there has been growing interest in the use of automated motion-sensing approaches for construction risk assessment.

Advancements in wearable sensors (WSs), particularly wearable inertial measurement units (IMUs), facilitate human-motion data capture in an effective and efficient manner. IMU sensors are widely used in commercial wearable products for health monitoring. A recent review by Yoong et al. [4], shows that, for posture, these products focus mainly on recognizing daily living postures related to spine movements based on sensor output. The limited posture monitoring capabilities of these products generally do not cover construction workers’ full-body awkward postures. In addition, commercial products typically provide diagnostic information (e.g., warnings and health scores from accompanying applications) without raw IMU output for customized posture analysis.

Closely-related studies have explored automating the detection of diverse awkward postures through the use of raw sensor output from both wearable IMUs [5,6,7] and smart-device built-in IMUs [8,9,10,11,12]. Existing studies mainly focus on optimizing the models for analyzing motion data and accurately detecting targeted human activities/postures [13]. Our review of closely-related studies (see Table 1) established that the application of machine-learning (ML) models for recognizing injury-prone postures is an active area in terms of IMU-based posture analysis for construction workers. Nevertheless, it is important to note that posture recognition is typically the first step in the real-world application of a WS system [14]. The captured postures should be appropriately analyzed and transformed into diagnostic information, which can then be provided to targeted end users as actionable feedback enabling behavior change for healthy outcomes [15].

In this sense, a fully functioning WS-based MSD-prevention system should integrate sensors applicable for workplace motion data collection; posture recognition and analysis models for injury risk assessment; and interfaces for communicating with end users (construction workers and safety managers in this context). The review of the closely-related work summarized in Table 1 shows that related studies lack the consideration of (i) MSD risks assessment based on captured postures; (ii) effective user interface (UI) design to provide feedback and intervention for proactive injury prevention; and (iii) users’ assessment of the WSs and UI that they directly interact with. Therefore, the goal of this study is investigating the design of an effective wearable MSD-prevention system for construction workers.

This study proposes a wearable sensing system that integrates IMU sensors for motion sensing, a deep neural network (DNN) model for posture recognition, posture-based ergonomics assessment models for MSD risk assessment, and UI for risk assessment feedback. A low-fidelity WS prototype was developed and experimentally used by construction workers performing routine construction tasks. Exemplary injury risk assessment feedback was then presented to workers and managers via the developed UI. Finally, the workers and managers who participated in the study were invited to complete a system evaluation survey, which assessed the proposed system regarding the usefulness and applicability of the proposed prototype. The results show that (i) the proposed WS system is a promising approach for collecting data from construction workers because it is not perceived to cause discomfort; (ii) the resulting posture-based MSD risk assessment information has a high potential for improving the workers’ safety awareness; and (iii) the developed mobile and cloud-based UI can readily deliver actionable MSD risk assessment information to the targeted end users. This study’s contribution to knowledge is the design, development, and assessment of a WS-based MSD prevention system for use in construction. Findings from this study can also inform the design of health monitoring systems in other labor-intensive sectors such as manufacturing, healthcare, and agriculture.

## 2. Methods

### 2.1. Prototype Design and Development for Wearable IMU Sensing System

This section describes the prototype design and development for a wearable IMU-based MSD prevention system. The prototype includes (i) a wearable IMU sensing system for posture recognition; (ii) posture-based ergonomics-assessment models for MSD risk detection; and (iii) the UI design for communicating MSD risk-assessment feedback to each worker and construction managers. The proposed prototype design is directed at effective MSDs prevention on construction sites. The following subsections first outline the design criteria and metrics for system evaluation (Section 2.1.1) and then describe the design and development for each sub-system (Section 2.1.2, Section 2.1.3, Section 2.1.4 and Section 2.1.5).

#### 2.1.1. Design Criteria and Metrics for System Evaluation

The system design criteria are related to each component of the proposed system design with corresponding metrics outlined in Table 2. The criteria and metrics were used for guiding the system design. Each metric was converted to question items in the end-user survey for system evaluation, which are discussed in detail in Section 2.2.

#### 2.1.2. Wearable IMU Sensing System for Posture Recognition

- Wearable IMU Sensor Selection

**Figure 1:**
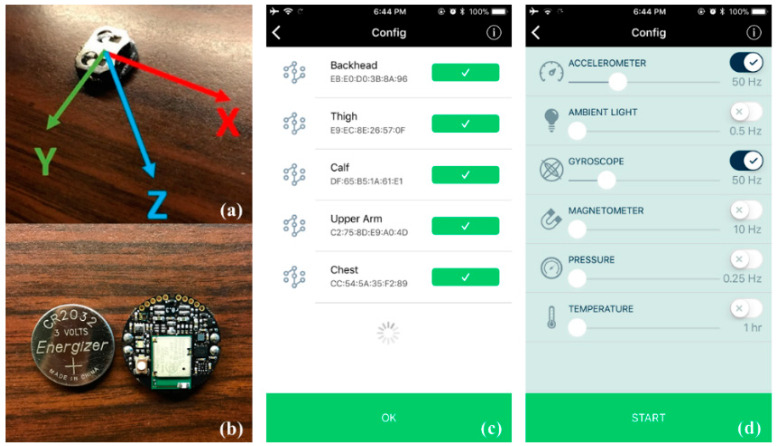
MetaMotion C sensor and interfaces [5]: (**a**) MetaMotion C IMU sensor board with the positive direction in each axis, (**b**) sensor board and battery, (**c**,**d**) are the MetaBase interfaces for configuring sensors on a smartphone.

The MetaMotion C sensors [18] were identified as a promising low-cost (US $75 each) IMU sensor for collecting motion data in a non-intrusive manner. Moreover, the sensor has an open-source application programming interface (API) for customizing the real-time data analysis programs. MetaMotion C can record the data to its onboard flash memory. It also has an included software package, MetaBase, which serves as the sensor controller interface, and can format the logged data into a .CSV file and export the data for analysis. MetaBase enables making simultaneous connections with multiple sensors. The sensors and UI are shown in Figure 1.

- Sensor Deployment

**Figure 2:**
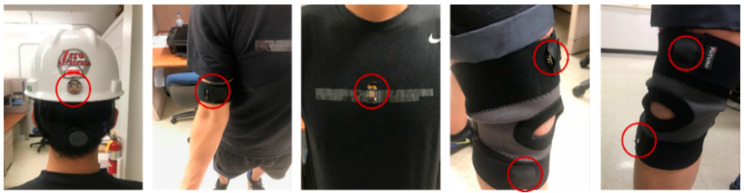
Sensor placement (head, right arm, chest, right thigh, and calf) in lab test [5].

**Figure 3:**
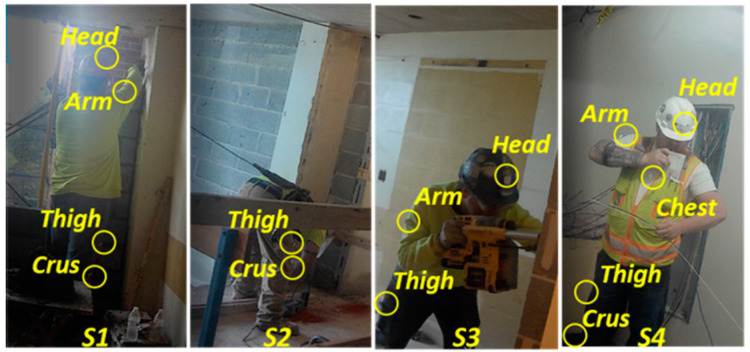
Subjects working with sensors (the sensors blocked are not circled) [17].

Sensor placement has a significant impact on posture recognition performance [19] and is correlated to the postures of interest for recognition. Sensors can be placed on the body parts corresponding to targeted postures, such as a head sensor for neck movement [20]. Previous studies placing IMU sensors around similar body landmark locations (as defined in Plagenhoef, et al. [21]) also showed that it was feasible to use this approach to capture motion data patterns characterizing workers’ postures [7,22]. Yan et al. [22] and Chen et al. [7] demonstrated the use of a full-body sensor placement approach using 17 IMU sensors. Our study selected sensor placements at the thigh, calf, chest center, upper arm, and head areas to limit the computation requirements and intrusiveness of full-body sensor placements. To further reduce the number of sensors used, the IMU sensors on leg and arm were attached only to one side (e.g., the right side of a right-handed person). A total of five sensor placements were selected in this study. The selective five-sensor placement has shown feasibility in capturing motion data for detecting construction-related postures in the authors’ previous experiments in both laboratory (Figure 2) and construction jobsite (Figure 3).

- Posture Recognition Models

Posture recognition from wearable IMU output is typically formulated as a classification problem, tasks for which ML-based models are receiving increasing research interest [17]. Applying conventional ML models (e.g., Support Vector Machine-SVM in Table 1) for recognizing workers’ postures and activities have also shown relatively high accuracy (within the range 60–90%) in related studies [5,6,7,8,9,10,11]. Applying deep learning (DL, a branch of ML [23]) models have achieved state-of-the-art accuracy when detecting human daily living activities from wearable IMU sensors [24,25,26]. 

**Figure 4:**
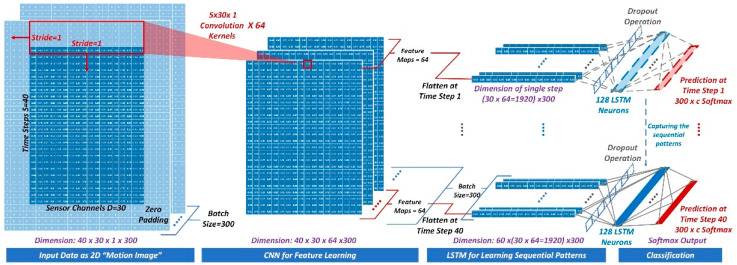
CLN conceptual architecture integrating one-layer CNN and one-layer LSTM. The detailed model parameter setup and evaluation is discussed in the authors’ previous work [17].

The successful deployment of DL models with multi-channel IMU outputs also drives their adoption for detection of construction activities and postures. Recent studies in construction demonstrate that using DL-based models has the potential for improving ML model performance in recognizing worker posture. For example, the long short-term memory (LSTM) model achieved accuracy up to 94.7% in Kim and Cho’s test [16]. Their previous experiment also showed that integrating convolutional neural network (CNN) and LSTM models as a convolutional LSTM (CLN) model can improve the accuracy of SVM (a recommended conventional ML model for posture recognition) by 3% [17]. In addition, the DL models developed using the featured framework (such as TensorFlow) can be readily migrated to a mobile device, which can further facilitate deploying an end-to-end posture analysis application on a smartphone. In this sense, this study adopted the previously developed CLN model (see Figure 4) for the proposed wearable IMU sensing system.

#### 2.1.3. Posture-Based Ergonomics Rules for MSDs Risk Assessment

**Figure 5:**
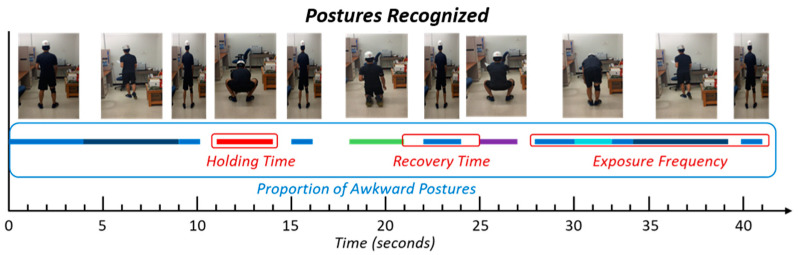
Posture recognition model output.

The output of posture recognition models includes detected postures with the timestamp of posture occurrence for each worker (see Figure 5 as an example). The following information can be extracted from the output, which was used for MSD assessment of individual workers: construction trade, types of awkward posture, holding time of static posture, the proportion of awkward work time, and distribution of awkward posture over time.

- Identification of Applicable MSD Risk Assessment Rules

To link detected postures with MSD risks, this study reviewed the commonly used ergonomics assessment rules using postures and timing information for injury risk analysis (see Table A1). The Ovako Working Posture Assessment System (OWAS) [27] and Maximum Holding Time (MHT) [28] rules were selected for MSD risk assessment considering the following:

(i) OWAS and MHT are two of the few rules that utilize specific evaluation criteria for predefined postures and provide information on any corrective actions to be taken. These not only enable the quantitative assessment of injury risks but can also provide actionable insight for injury prevention responses.
(ii) Both OWAS and MHT quantify human postures via rough relative positions between different body segments, instead of direct measurement (for body segments deviation or joint angles). The posture recognition models in this study were trained to recognize working postures labeled by observation (not precise body segments measurement). They are, therefore, suitable for use in risk assessment using the recognized postures.
(iii) The thresholds provided in OWAS and MHT can be used for tracking MSD risks in real time and triggering effective intervention on time.
(iv) In terms of the validity of ergonomics risk assessment, the OWAS has demonstrated high repeatability and association with MSDs in cross-correlation studies [29]. The MHT criteria, developed from epidemiology studies, also show high validity when compared with discomfort levels.

- Real-Time Maximum Holding Time Assessment for Individuals

The real-time MHT assessment is directed at reducing overexposure to prolonged static awkward postures. The continuous holding time of certain postures can be captured and compared with the corresponding assessment threshold. Once the safety threshold is breached, warning signals can be triggered to make a posture correction recommendation. The developed model uses motion data collected for every second to recognize the corresponding postures. The breach of the MHT threshold can be detected in real time by counting the number of consecutive identical postures and comparing it with the corresponding threshold. A real-time warning for individual workers can be sent for timely posture correction once the threshold is exceeded. The algorithm’s pseudo-code for the assessment program is presented in Table A2.

- Periodic MSD Risk Assessment for Individual

A periodic assessment was performed to evaluate an individual worker’s risk of MSD during a unit of working time. A posture profile can be built through recognizing all the postures from a span of working time (e.g., 30 min). The MSD risk is evaluated by comparing the captured postures with thresholds. Table 3 presents the information captured periodically from the posture recognition model and applicable MSD assessment. The output from the periodic assessment can be used as feedback to workers about their MSD risk. This keeps them informed about potential risks accrued from overused postures. This information can also be packaged into recommendations for posture correction. The algorithm pseudo-code for the periodical assessment program is presented in Table A3.

- Periodic MSDs Risk Assessment for Jobsite

The MSD risk assessment for the jobsite was designed for identifying construction trades with the highest MSD risks and the cumulative time of high-risk overexposure during one workday. The jobsite-level assessment was based on synthesizing the individual worker’s posture recognition results from different trades over time. A 30-min interval was used as an example to summarize all the postures recognized from each trade. The participants were from different trades. The average number of postures (for one worker in a specific trade) was obtained for comparing the awkward postures among trades. Table 4 shows the information used for jobsite MSD risk assessment.

#### 2.1.4. Mobile Application UI for Delivering Individual Assessment Information

**Figure 6:**
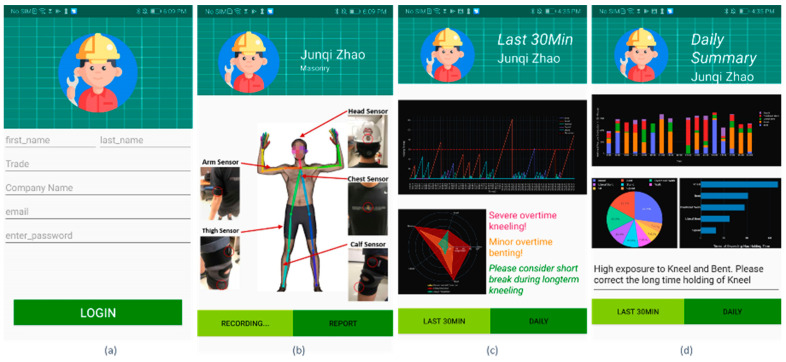
Mobile application UI, which includes: (**a**) login interface, (**b**) sensor placement instructions, (**c**) 30-min assessment feedback, and (**d**) daily assessment feedback.

A mobile application UI, developed for the Android operating system, was deployed to communicate the results of MSD risk assessment result to each worker (Figure 6).

The UI includes the following features: a login interface, sensor placement instructions, a 30-min assessment feedback, and a daily assessment feedback. The assessment results were based on exemplary posture data for demonstration purposes. Each worker is required to type in their user information before logging into the application (Figure 6a). This is designed for collecting worker’s trade information for further jobsite-level assessment. After logging into the application, there is an instructions page (Figure 6b) that shows how to place the sensors properly.

The assessment summary (Figure 6c) is provided after every 30 min of work and includes: (i) posture profile for the last 30 min; (ii) postures held for too long; and (iii) overused postures and suggestions for correction based on ergonomic rules. Similarly, the daily summary (Figure 6d) reports: (i) distribution of awkward postures over time, (ii) proportion of different award postures, (iii) ranking of postures breaching MHT thresholds, and (iv) suggestions for further posture correction. The source code for the UI design is available online [30]. This study aims to proactively intervene in the development of MSDs by delivering the individual-level assessment information. Specifically, the real-time alarm can be used for the timely correction of prolonged awkward postures. The proposed periodic assessment can improve the targeted workers’ awareness of the awkward postures and encourage them to adopt self-correction practices to reduce the risk of developing MSDs in the long term.

#### 2.1.5. Cloud-Based Dashboard UI for Delivering Jobsite Assessment Information

**Figure 7:**
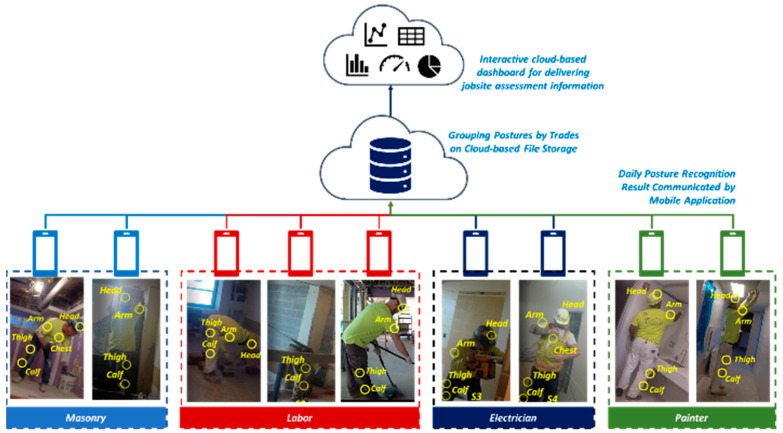
System architecture of the MSD assessment information delivery system.

The MSD risk assessment information on jobsites is provided to the construction managers so they can make a holistic evaluation of and mitigate potential MSD-related injury risks. A cloud-based dashboard prototype was proposed to synthesize and deliver the jobsite assessment information. Figure 7 depicts the system architecture of the cloud-based dashboard.

The jobsite-level assessment information is designed to be delivered on a daily basis. Captured postures from each workers’ daily work are stored on their smartphones. Daily posture recognition results are uploaded to a cloud-based file management system, such as Google Drive, for further processing. Each worker’s trade information is recorded when they log into the application. Individual workers’ postures are grouped by trade without revealing identifiable personal information. The deployed “group-by-trade” process was designed to be implemented via the cloud-based file system. The captured postures from all trades are synthesized and reported via a cloud-based dashboard from a webpage. 

**Figure A1:**
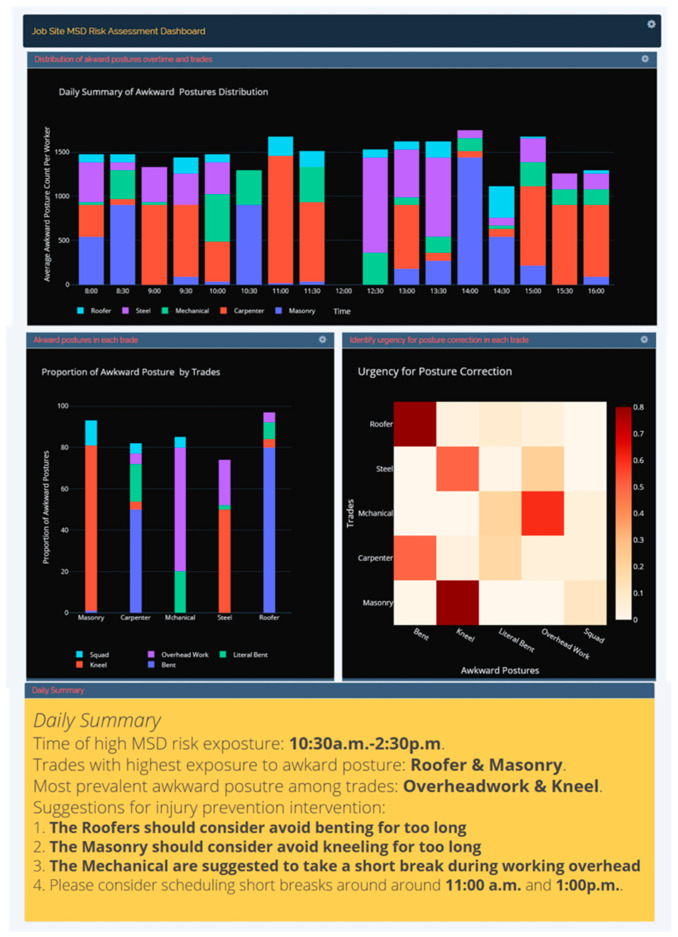
Cloud-based dashboard interface.

Our proposed cloud-based dashboard prototype was developed using Plotly Dash (https://plot.ly/dash/ (accessed on 28 November 2019)), which visualizes assessment results as an interactive dashboard for checking detailed assessment reports. An exemplary demonstration dashboard was created (see Figure A1). The delivered assessment information was built on the synthesizing strategy presented in Table 4. The following detailed assessment information was delivered to the construction safety manager via dashboard: (i) awkward posture distribution over time and trades; (ii) proportion of awkward posture by trades; (iii) urgency for posture correction among trades; and (iv) daily summary and safety recommendations. The interactive UI example is available online [31].

### 2.2. End-User Evaluation Survey 

The targeted end-users (construction workers and managers) were invited to interact with the wearable IMU sensing system and UI prototype that was developed. Feedback on their experience was captured via a structured survey to evaluate the usefulness and applicability of the proposed prototype. The following describes the survey design and implementation procedures.

#### 2.2.1. Design of the Evaluation Survey

We collected demographical information regarding trade of worker; job position of manager; years of experience in current trade or position; and work-related injuries of workers in the past five years. Based on the design criteria and metrics proposed in Table 2, a structured survey questionnaire was designed using five-level Likert scale questions (see Table A4). This questionnaire was used to collect the subjects’ evaluation and perception of the design of the proposed prototype. The survey questions were set up to evaluate the performance of the wearable sensing system with respect to MSD risk assessment and the delivery of information to workers. The questions were directed at supporting the participants to perform a holistic evaluation of system usefulness, applicability, and acceptance. The questionnaire also included open-ended questions allowing the participants to explain the reasoning behind their responses. The participants were also invited to provide feedback and suggestions for system improvement.

#### 2.2.2. Implementation of the Evaluation Survey

**Figure 8:**
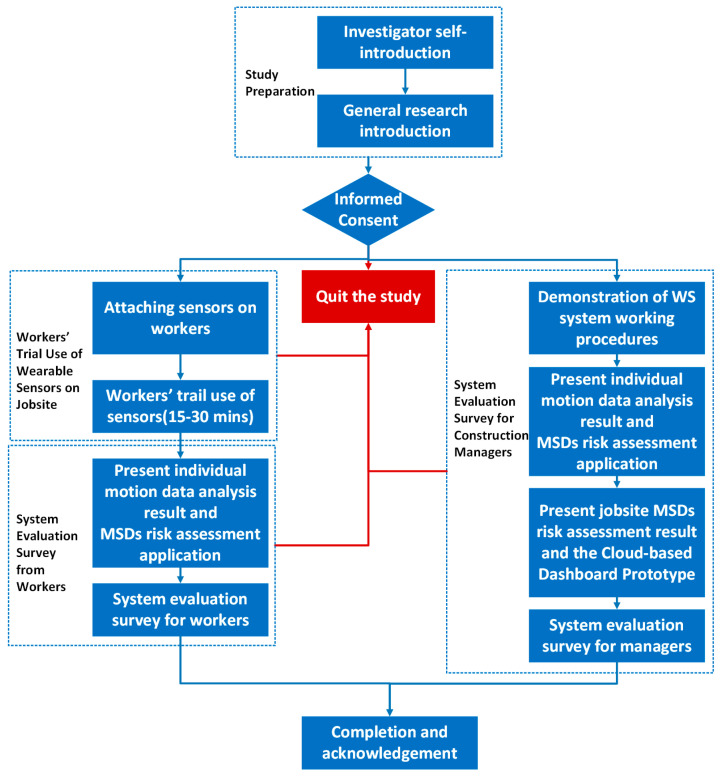
Implementation procedure for evaluation survey.

We recruited both worker and manager subjects from a residential building project under construction on the University Park campus of The Pennsylvania State University. Figure 8 outlines the procedure used to conduct the survey.

Construction workers participated in the following major steps:

Step 1: Workers were first invited to a trial use of wearable IMU sensors during their daily tasks for 15 to 30 min with video recording. Five sensors were then deployed on the surface of the workers’ clothes and hardhat (as shown in Figure 3) after obtaining the workers’ consent. The workers then conducted their routine tasks for 15 to 30 min while wearing the sensors.
Step 2: The individual-level motion data analysis and MSD risk assessment results were presented to each worker. Each worker experienced interacting with the mobile application UI (see Figure 6). In this case, the workers obtain a better understanding of the content of individual-level MSDs assessment information, approaches for information delivery, and how the assessment information can help them prevent injuries.
Step 3: The evaluation survey was then conducted for each worker.

Construction managers (including construction engineers and management personnel) from the project management team were invited to participate the following steps:

Step 1: All managers were invited to join the evaluation survey session through a meeting room on the jobsite.
Step 2: The wearable IMUs sensing system prototype was presented to the managers in detail. They watched recorded videos of workers wearing the sensors while they performed their routine tasks. This helped the managers understand how the WS-based motion data collection system works on jobsites. They were also shown the deployed mobile application UI demonstrating how our approach can be used to deliver individual MSD risk assessment information. The managers accessed jobsite-level MSD risk assessment information through interaction with an online dashboard. They were also presented with a demonstration of the potential application of the proposed approach as a decision support system for construction health and safety. This process was designed to enhance the construction managers’ understanding of how the proposed system could be implemented for use in MSD prevention.
Step 3: The managers were invited to complete the evaluation survey.

## 3. Results and Discussion

This section presents and discusses the results based on the targeted end-users’ evaluation of the proposed system. Posture recognition performance of the proposed CLN model is also discussed. 

### 3.1. Summary of the Demograhical Profile of the Subjects

**Figure 9:**
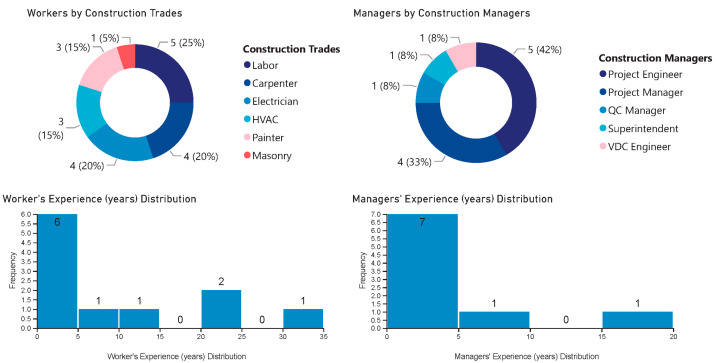
Description of survey subjects.

Thirty subjects enrolled in the evaluation survey, a mix of 18 workers and 12 managers. Figure 9 describes the distribution of subjects with respect to trades, positions, and years of working experience. Subjects’ responses (median of response) were summarized based on the previous Likert scale. The Wilcoxon Test [32] was applied for statistical inference analysis, considering (i) the subjects provided an ordinary response (one to five) in the survey; and (ii) the sample size for both worker (18) and manager (12) are relatively small. In this sense, the Wilcoxon Test is appropriate, given that the subjects’ responses may not satisfy the normality assumption [33].

### 3.2. Workers’ Evaluation of Wearable Sensing System

Table 5 presents the analysis of all subjects’ responses to the wearable sensing system evaluation.

#### 3.2.1. Comfort

The captured physical and mental discomfort ratings reflect the workers’ perceived level of physical inconvenience when using WSs, e.g., a high rating represents a high level of discomfort. Results presented in Table 5 show that: (i) both physical and mental discomfort ratings were significantly lower than a moderate level, except that manager subjects expected a moderate level of mental discomfort for workers, and (ii) both physical and mental discomfort ratings of workers were significantly lower than those of the managers. The results suggest that workers believe that using WSs during construction tasks cause a low level of discomfort. Workers who experimentally used the sensors tended to feel a lower level of discomfort than what the construction managers expected. 

We also compared the responses from the workers and managers for the open-ended questions to examine their perception about the sensors. The miniaturized size of IMU sensors was unlikely to cause severe physical and mental discomfort, according to the managers’ response. However, the managers also raised concerns over the mental discomfort of workers as they might feel that they were being monitored. They might also be distracted by the fears that sensors could fall off if the attachment was loose. Educating the workers on the use of the sensor system could help with reducing their mental stress, as suggested by one manager. One worker also mentioned that understanding the reasons for using the sensor system could help avoid mental discomfort. In summary, the proposed wearable sensing system elicited a low level of mental and physical stress for workers. Training or education for workers could alleviate potential mental stress when using the sensor system.

#### 3.2.2. Intrusiveness, Inconvenience, and Durability

The intrusiveness and inconvenience metrics measure the potential adverse effects on the performance of workers’ daily tasks. The results show that workers rated the level of intrusiveness and inconvenience as low. The workers indicated that the sensors did not greatly lower their productivity because they can still do their daily tasks. Nevertheless, two workers also mentioned that certain sensor placement, such as those placed at chest center (interfering with lifting task) and knee areas (interfering with the use of knee pads), might interfere with their daily tasks.

In terms of the sensors’ durability to the harsh jobsite environment, workers who experimentally used the sensors gave significantly lower ratings than construction managers. Seven workers and three managers indicated that they had some concerns that the sensors (currently attached by hook-and-loop tape) might fall off and go missing, which would be inconvenient and decrease durability. Suggestions from the subjects show that such concerns could be addressed through better attachment, such as pining, clipping, and wrapping the sensor onto personal protective equipment (PPE).

#### 3.2.3. Acceptance

Table 5 shows that both workers and managers gave a moderate rating for the level of acceptance of WSs on jobsites. The moderate level of acceptance was further examined through the subjects’ responses to open-ended questions. Two workers indicated that they were willing to wear the sensors if needed or as required. Two workers also expressed concern that the sensors could get lost or broken, which could be an issue for user acceptance. The managers participating in the study suggested that user acceptance levels could be higher if workers keep using the sensors and get used to them. These results suggest that: (i) the WS durability and robustness, particularly the sensor attachment method, should be discussed further with both the workers and managers to increase acceptance levels; (ii) the results obtained from the interactions with the end-users should be used to develop and deploy education and training programs directed at motivating workers’ interest in WS technology–based safety management. Such an approach will ensure that targeted workers are aware of the reason and importance of using this approach to MSD injury prevention, which will, in turn, increase the adoption rates for the proposed WS-based safety management practices.

### 3.3. Evaluation of Injury Risk Assessment and UI 

Table 6 describes all subjects’ responses to the content of injury risk assessment, which is discussed further in the following sections.

#### 3.3.1. Content of Assessment Information

Usefulness of immediate intervention: The trigger for immediate intervention in our proposed injury prevention approach is through a real-time alarm notification to workers who are overexposed to high injury risks (e.g., breaching MHT). Both workers and managers gave a high usefulness rating for such intervention. The managers observed that such an approach could help with the difficult task of tracking bad postures and enhancing the workers’ awareness of potential injuries, thus taking preventative actions (e.g., taking a short break). The workers also indicated that the alarm for intervention was useful for correcting bad postures. However, workers also raised two practical concerns: (i) not everyone carries smartphones during their work for accessing the assessment information and (ii) frequent false alarms from errors in posture detection can be annoying.

Usefulness of periodic assessment: Both worker and manager subjects gave a high usefulness rating for the periodic assessment report on awkward postures at the individual or entire jobsite, which as designed can be delivered either every 30 min or daily. Two workers indicated that this would allow them to know risky postures that can have a bearing on their health in the long term and understand how they can adjust their postures to prevent injury. One manager commented that the time profile of awkward postures on the jobsite could help with safety planning. Moreover, knowing the trades with high awkward postures exposure and types of these postures can help a construction company conduct proactive injury prevention, through, for example, implementing safety equipment for targeted trades. Managers also expressed an interest in periodic assessment across longer time periods (e.g., weekly reports) as this allows them to track tracking long-term behavior patterns.

#### 3.3.2. User Interfaces for Delivering Assessment Information

Mobile application: The mobile application UI was designed to communicate (i) the incidence of recurring awkward postures (every 30 min or daily), (ii) the daily time profile of awkward postures, and (iii) the need for taking immediate intervention with the workers. Both workers and managers gave high usefulness ratings for the mobile UI. The subjects mentioned that this could help workers correct their bad postures and prevent potential injuries over the long term, and it is particularly useful for workers who already have chronic injuries. One worker also shared that his previous experiences with wearable sensing products and mobile applications made him willing to adopt using the mobile UI.

Notably, the workers gave a significantly lower usefulness ratings for immediate intervention (with a median rating of level 3) as compared to the other two pieces of information (both with a median rating of level 4) delivered on the mobile UI. Two considerations were listed by the workers to support their lower ratings for real-time alarm via the smartphone: (i) not every worker carries a smartphone during work and (ii) there are some postures that workers cannot change for the task—it is the way they are done. One manager also mentioned that workers tend to ignore the alarms if there were too many. A real-time alarm might not work effectively in these situations. Providing a record of awkward postures periodically to workers might be a better option.

On-line dashboard: The usefulness of the assessment information delivered via the on-line dashboard was perceived to be high. Managers had a higher preference for knowing awkward postures distributed among trades and their associated risk levels (with the median rating of level 4.5). As discussed previously, knowing these can help with better safety planning and supporting company-level safety decision making.

#### 3.3.3. Frequency of Delivering Assessment Information

How frequently the assessment information should be delivered to users is an important consideration when designing an effective feedback mechanism. We compared the ratings for different frequencies of feedback given to both workers and managers. The managers perceived the jobsite-level assessment information delivered daily as highly useful. Both workers and managers gave a higher rating for daily information delivery and a lower rating for a frequency of every 30 min. One manager commented that delivering an assessment report every 30 min was too often and that workers will not look at it. The workers’ responses also showed their preference for a daily assessment summary delivered at the end of every day’s work shift. These re-affirm the evaluation results in the preceding sub-section. Although real-time or frequent assessments may provide more timely feedback and intervention, the end users prefer to be presented with assessment feedback after finishing their work shift, instead of being interrupted too often by feedback during their shift. Additionally, both workers and managers showed a preference for additional weekly assessment summaries. This can help with tracking long-term trends of awkward posture exposure and correction.

We observed a consistently low rating for usefulness or preference for real-time injury risk warning, feedback, and intervention when evaluating mobile UI and information delivery frequency. Real-time feedback was proposed as a proactive intervention in the cumulative injury development process. Providing timely intervention is theoretically valuable if the workers make immediate responses after receiving warning feedback, as assumed. However, there exist practical issues for deployment in the workplace, such as access to smartphones (which deliver feedback warnings). Moreover, some psychological factors should also be considered. Warnings that are perceived as being too frequent can be annoying and are most likely going to be ignored by workers, as mentioned by the subjects of the survey. 

A periodic assessment summary (on a daily and even weekly basis) can better serve as the facilitator of behavior change and proactive injury prevention. The daily risk assessment information after a work shift can be well perceived by workers, thus improving safety awareness and instigating behavior modification over time. It is worth noting that workers who are constantly subjected to increased pressures for productivity improvement are more susceptible to adopting awkward working postures. These observations and findings can be used as guidance for developing a behavior intervention approach for increasing productivity on the jobsite in a way that also improves the health and safety of the workers.

### 3.4. Holistic System Evaluation

Table 7 describes the holistic evaluation of the perceived usefulness, applicability, and likelihood of adopting the proposed wearable IMU sensing system for MSD prevention among workers and managers.

#### 3.4.1. Potential System Usefulness

Both workers and managers rated the usefulness of the proposed system as high. The managers perceived the proposed system to be a good alternative approach for safety monitoring. Because it allows gathering health and safety-related data for proactive prevention, instead of relying on manual observations, the managers also mentioned that the captured information could improve the workers’ awareness of their postures and train workers for self-correction, thus preventing injuries over the long term. Furthermore, reducing employee injury was also perceived as a beneficial undertaking for a construction company, considering that this can show the company’s care for workers’ health and reduce insurance costs over the long run. 

Notably, both workers and managers mentioned a common potential issue limiting the effectiveness of the proposed system. The WS system and delivered assessment information may affect the workers’ productivity and progress. The productivity pressure may require workers to return to awkward postures. Further longitudinal studies should be done to investigate whether a worker’s productivity will be affected when they use WSs during daily tasks.

#### 3.4.2. System Applicability

The managers were asked to evaluate the system’s applicability for deployment on the construction jobsite. The results show an applicability level between moderate to high. The most salient issue with system application is the sensor durability on construction sites based on the subjects’ responses. The subjects mentioned that sensors should be both durable and reusable for construction workers. Such results are consistent with the feedback from workers. Addressing the observed concerns over the durability of the sensor (e.g., attachment method) is a critical factor for the successful deployment of the system on the construction site.

#### 3.4.3. Likelihood of Adoption

The results also showed a moderate level of system adoption among workers and managers. The following issues were identified via the survey: (i) sensor robustness/durability to harsh conditions (e.g., tight working spaces); (ii) sensor interference with other safety equipment (e.g., harnesses and knee pads); (iii) workers’ accessibility to a smartphone during work; (iv) cost (e.g., who should pay for the sensors); and (v) awareness of injury risks from awkward postures—some subjects do not think awkward postures are risk factors for injuries. Despite the high perceived system usefulness, further work is required to increase system adoption. Sensor durability should be enhanced for use on construction sites, such as being integrated into PPE so as to not interfere with other safety equipment. Moreover, both construction workers and managers should be aware of the risks of overexposure to awkward working postures. Health and safety education is helpful in building a mindset for preventing long-term injuries via a proactive approach.

### 3.5. Performance Evaluation of Posture Recognition Model

#### 3.5.1. Description of Collected Motion Data

Workers’ postures were video-taped as the ground-truth reference during their experimental use of WSs. The video reference and sensor output were synchronized after data collection. Then, we labeled each subjects’ motion data with corresponding working postures using video reference. Table 8 presents a summary of all the labeled postures from 19 subjects. In addition to the 18 worker subjects that participated in the survey, another worker joined the motion data collection without participating in the survey. The motion data used for testing the posture recognition model were collected from a total of 19 workers.

#### 3.5.2. Test of Posture Recognition Model

**Figure 10:**
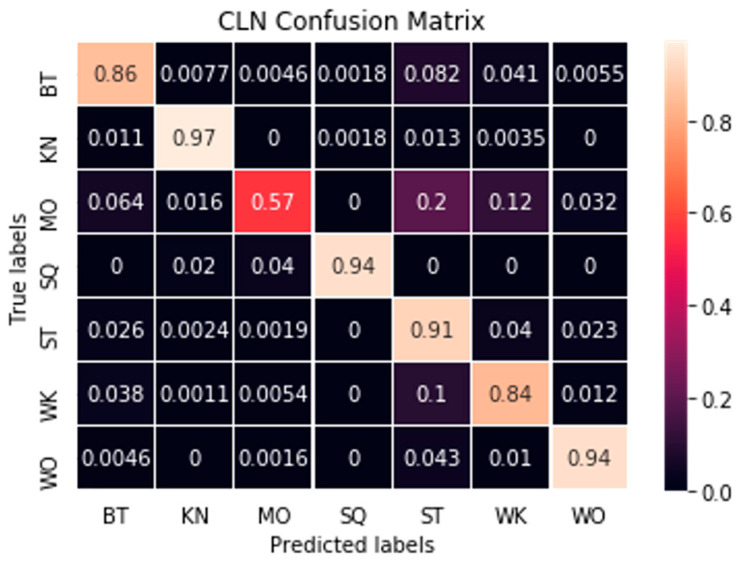
Confusion matrix of posture recognition. The numbers were normalized for better interpretation.

The CLN model proposed in our previous work [17] was adopted in this study as the recognition model. The CLN model comprised one convolutional layer and two LSTM layers, which has shown promising recognition performance in our experiment with four workers [17]. This study further tested the model performance using posture data from 19 workers during their daily tasks. Specifically, all the 19 subjects’ posture data were combined and randomly split as training (72%), validation (18%), and testing (10%) subsets. We repeated the test for five rounds with different random seeds to obtain the average model performance. The model performance test results and confusion matrix are provided in Table 9 and Figure 10.

Results in Table 9 show an overall recognition accuracy of 0.9. Such a result indicates that 90% of the postures performed by the subjects can be correctly recognized by the proposed CLN model. The awkward postures (such as BT, KN, SQ, and WO) were also detected with relatively high F1 scores (all over 0.9 except for 0.86 for BT), which suggest promising results for detecting these injury-related postures, despite that they were the minority in the collected postures. A close examination of recognition errors (in Figure 10) shows that the BT posture tended to be misclassified as MO. One possible reason is that the MO comprised a series of postures, such as BT, ST, and SK, when workers climb up and down, which may lead to the misclassification between BT and MO. Additionally, the limited MO data (0.8% as show in Table 8) may also impede adequate learning and result in high recognition errors of MO.

#### 3.5.3. Examination of Effective Sensor Position and Channels

**Figure 11:**
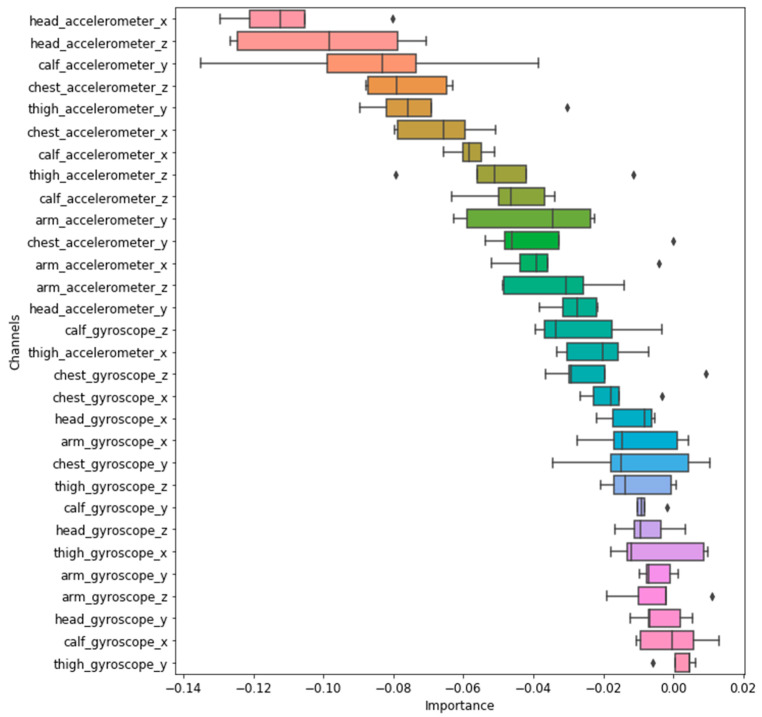
Ranking of feature importance.

This study further examined the effective sensor placement and channels for posture recognition with the proposed CLN model. Direct interpretation of features (i.e., sensor output from different positions and channels) used in the DNN-based model is challenging as feature learning is treated as a “Black Box”. We, therefore, applied the “Feature Permutation” method to evaluate the importance of different sensor channels played in the proposed CLN model. A feature is “unimportant” if shuffling its values leaves the model error unchanged, as the model ignored this feature for the prediction [34]. Each sensor channel within the test subset was shuffled before feeding into the trained CLN model. The performance reduction (from the CLN model using unshuffled test data) was used for evaluating feature importance. The test was also repeated for five rounds. Figure 11 describes the ranking of feature importance, where the order was based on the average importance from the five-round test.

Results in Figure 11 show that the top 14 important features were based on accelerometer output. The last 14 features were from the gyroscope. These suggest that accelerometers contribute more to characterizing workers’ postures in daily tasks. In terms of sensor positions, the accelerometers placed at the head and calf tended to have a higher impact on the CLN model’s performance. The arm sensor imposed less influence on posture recognition results. The improper sensor placement may not provide useful information for the recognition models to correctly recognize the postures and be noise for models. Moreover, the increased number of sensors also brings more data for processing, which leads to increased computational complexity and implementation cost. These results may help to guide the wearable sensing system design by further reducing the number of required sensors with marginal impact on posture recognition performance.

## 4. Conclusions

Advancement in wearable sensing greatly facilitates workplace safety monitoring and data-driven injury prevention. This research presents the design of an effective wearable MSD prevention system for use in the construction industry. This study first proposes a wearable IMU sensing system, with a prototype developed for end-user evaluation. Construction workers and managers evaluated the proposed wearable IMU sensing system through interacting with the WSs and a UI. Then they participated in an end-user survey. The following findings were obtained from end-user evaluation regarding the proposed system design.

A WS system is a promising approach for collecting data from construction workers because it is not perceived to cause discomfort. Although one respondent indicated that there was value in avoiding interference with safety equipment (e.g., knee pads), the workers did not consider the proposed use of WSs as being intrusive. Sensor durability/robustness is critical to further improving its applicability and acceptance on jobsites. 

The resulting posture-based MSD risk assessment information has a high potential for improving the workers’ safety awareness. An improved awareness about exposure to awkward postures can potentially trigger posture correction by workers. The jobsite risk assessment can help managers make more informative health and safety decisions by for example improving safety planning and implementing specific safety equipment for different construction trades. 

The developed mobile and cloud-based UI can readily deliver the MSD risk assessment information to end users. A daily report is preferred among end users for accessing assessment information, considering that: (i) workers might ignore alarms and assessment provided at a high frequency, and (ii) not every worker carries a smartphone for accessing alarms during daily tasks.

The proposed convolutional LSTM model shows a high potential in automated recognition of workers’ postures in daily tasks. It is also important to note the insufficient training dataset and concurrent postures (such as multiple postures involved in climbing) may impair model performance. Additionally, accelerometers tend to be more effective for characterizing workers’ postures than the gyroscopes. Head and calf areas can be effective placement for posture recognition.

Our proposed wearable IMU sensing system for proactive MSD prevention has high potential for reducing construction workers’ risk of developing awkward posture-related injuries over the long-term, which, in turn, benefits the employer due to reduced compensation costs and demonstrating care for workers’ wellbeing. Continued use of the proposed system can be achieved by addressing some concerns about sensor durability, the risk of adverse effect on the workers’ productivity from the proposed approach, and the overall cost-effectiveness of potential MSD mitigation strategies.

## 5. Limitations and Future Work

One limitation of this study is the relatively low number of participants in the evaluation survey for the proposed wearable system. Worker subjects also experimentally used the sensors for only 15 to 30 min while performing routine tasks. Wearing the sensors for longer sessions during workers’ daily shifts might provide more evidence regarding the usability of sensors. Further work can be done by recruiting more workers from different trades for extended trial-use sessions. Given that some trades are more susceptible to MSDs (e.g., back pain is highly prevalent among masons), further work can focus on expanding the sample size from these targeted trades. It is important to note that delivering the assessment feedback to workers aims at improving their overall safety awareness and, ultimately, instigating behavior-changes for reducing injury risks. Future research can compare the patterns of workers’ use of awkward postures before and after providing the injury risk assessment to them. The changing patterns of safety behavior can also be used to evaluate the effectiveness of construction safety management practices. 

## Figures and Tables

**Table 1:** Review of related studies applying machine learning (ML)-based models for inertial measurement unit (IMU) output in construction.

Models *	**Motion Data Collection**	**Placement** **(Numbers)**	**Recognition Performance**		**Risk Assessment and** **Feedback**
			**Classes**	**Accuracy**	
NN [8]	2 workers conducted prescribed activities in experiments	Arm (1)	3	~90%	N/A
SVM [6]	21 workers conducted prescribed masonry tasks in experiments	Full-body (17)	2	~91%	N/A
SVM [7]	4 students conducted prescribed awkward postures in experiments	Full-body (17)	9	~60–80%	N/A
SVM [9]	10 workers conducted prescribed masonry tasks in experiments	Wrist (1)	4	~88%	N/A
SVM [5]	1 student conducted prescribed award postures in experiments	Full-body (5)	7	~74–83%	N/A
SVM [10]	25 students conducted prescribed activities in experiments	Leg and wrist (2)	8	89%	N/A
SVM [11]	2 workers conducted prescribed activities in experiments	Arm and wrist (2)	3	up to 90%	OSHA Rules
LSTM [16]	3 students conducted prescribed activities in experiments	Hip and neck (2)	11	up to 95%	N/A
Convolutional LSTM [17]	4 workers conducted natural postures in daily tasks	Full-body (5)	8	85% (Macro F1 Score)	N/A

* The table features recognition models that achieved the highest recognition performance in the tests of corresponding studies. NN—Neural Networks, SVM—Support Vector Machine, LSTM—Long Short-Term Memory.

**Table 2:** System criteria and metrics for evaluation.

System Design Criteria	Metrics		
Wearable Sensing System	Physical Discomfort Level	
	Mental Discomfort Level	
	Inconvenience Level	
	Intrusiveness Level	
	Durability and Robustness Level	
	Acceptability of Wearable Sensors	
Risk Assessment and Information Delivery System	Content of Assessment	Usefulness of Immediate Intervention
		Usefulness of a Periodical Assessment
		Trust in Proposed Assessment
	Usefulness of Mobile App-based Assessment Information Delivery for Individuals	Incidence of the recurring awkward posture of individuals
		Daily time profile of awkward postures
		Need for taking immediate intervention
	Usefulness of Online Dashboard-based Assessment Information Delivery for Jobsite	Daily time profile of awkward postures
		Incidence of recurring awkward postures by trades
		Injury risk level by trade
		Recommended speed of taking intervention by trade
	Usefulness of Information Delivery at Varying Frequency	Real-time
		Every 30 min
		Daily assessment
Holistic Evaluation of System Design	Potential System Usefulness	
	System Applicability	
	Likelihood of Adoption	

**Table 3:** Periodic MSDs risk assessment for individual workers.

Captured Information	Risk Assessment of MSDs
Total count of posture *i* ^1^ breaching MHT	Informing the construction workers about their prolonged postures used in the unit working time interval. Measuring the repetitiveness of awkward posture in unit time. Note: No associated criteria or threshold for posture correction suggestion.
The total duration of posture *i* breaching MHT	
Max time of holding posture *i*	
Average frequencies of posture *i* occurrence in one minute	
The max holding time of posture *i*	
Proportion of posture *i*	Comparing with OWAS to assess risks of MSDs and infer the urgency for making corrections.

^1^*i* represents a certain posture.

**Table 4:** Periodic MSDs risk assessment on jobsites.

Synthesized Information	Risk Assessment of MSDs on Jobsite
A daily summary of awkward posture distribution Obtain the average number of detected awkward postures in every 30 min for each trade Combine the detected awkward postures from each trade in the same 30-min interval Extend the every-30-min assessment to one day’s shift	Identifying the time of the day with the highest overexposure to awkward postures.
	Identifying the trades with the highest number of awkward postures in a certain timespan.
	Obtaining a time profile of awkward posture distribution on construction jobsite.
The proportion of awkward posture by trade Obtain the proportion of awkward postures in one-day’s work Comparison of awkward posture proportion across trades	Identifying the construction trades with the greatest proportion of awkward postures on jobsite in one-day work.
	Identifying the awkward posture accounting for a high proportion of a worker’s postures.
The urgency for posture correction Obtain the proportion of awkward posture by trade Compare the awkward posture proportion with assessment rules (OWAS) for each trade	Identifying the awkward postures that need timely correction for mitigating MSDs risks for each trade.

**Table 5:** Evaluation of wearable sensors.

System Design Criteria	Metrics ^1^	Response (Median)	
		Worker ^2^	Manager ^2^
Wearable Sensing System (1—Very Low, 5—Very High)	Physical Discomfort Level ***	1 ***	2 **
	Mental Discomfort Level ***	1 ***	2 *
	Inconvenience Level	2 ***	N/A ^3^
	Intrusiveness Level *	2 ***	2 ***
	Durability and Robustness Level ***	3 *	3.5 **
	Acceptability of Wearable Sensors *	3 *	3 *

^1^ Unpaired Wilcoxon Test for whether the response is different between workers and managers for the same item. ^2^ Unpaired Wilcoxon Test for whether the subjects’ response is different from “moderate level (3)” for each item. ^3^ N/A denotes the question item was not provided in this group of subjects. *** 0.05 level of significance, ** 0.1 level of significance, * not significant at level of 0.1.

**Table 6:** Evaluation of risk assessment information and UI for information delivery.

System Design Criteria	Metrics ^1^		Response (Median)	
			Worker ^2^	Manager ^2^
Risk Assessment and Information Delivery System (1—Very Low, 5—Very High)	Content of Assessment	Usefulness of Immediate Intervention **	4 ***	4 ***
		Usefulness of Periodic Assessment	4 ***	4—Refine Schedule *** 4—Evidence-based Intervention ***
	Mobile App-based Assessment Information Delivery for Individuals	Incidence of the recurring awkward posture of individuals *	4 ***	4 ***
		Daily time profile of awkward postures *	4 ***	4 ***
		Need for taking immediate intervention ***	3 **	4 ***
		Rank of Preference ^3^	Incident = Profile > Immediate ***	All equal
	Online Dashboard-based Assessment Information Delivery for Jobsite	Daily time profile of awkward postures	N/A^4^	4 ***
		Incidence of recurring awkward postures by trades		4 ***
		Injury risk level by trade		4.5 ***
		Recommended speed of taking intervention by trade		3.5 ***
		Rank	Daily Profile = Incident = Risk Risk > Speed ***	
	Frequency of Information Delivery	Daily summary for jobsite	N/A	4 ***
		Real-time for workers ***	3 **	4 ***
		Every 30 min for workers **	3 *	3 *
		Daily assessment for workers ***	4 ***	4 ***
		Rank of Preference ^3^	Daily > 30 **	Daily = Real-time > 30 ***

^1^ Unpaired Wilcoxon Test for whether the response is different between workers and managers for the same item. ^2^ Unpaired Wilcoxon Test for whether the subjects’ response is different from “moderate level (3)” for each item. ^3^ Paired Wilcoxon Test for whether the subjects’ response is different for comparable items, e.g., whether the worker subjects prefer “Real-time warning” over “Daily assessment” in terms of information delivery frequency. ^4^ N/A denotes that the question item was not provided in this group of subjects. *** 0.05 level of significance, ** 0.1 level of significance, * non-significant at level of 0.1.

**Table 7:** Holistic assessment.

System Design Criteria	Metrics ^1^	Response	
		Worker ^2^	Manager ^2^
Holistic Evaluation (1—Very Low, 5—Very High)	Potential System Usefulness *	4 ***	4 ***
	System Applicability	N/A ^3^	4 **
	Likelihood of Adoption *	3 *	3 *

^1^ Unpaired Wilcoxon Test for whether the response is different between workers and managers for the same item. ^2^ Unpaired Wilcoxon Test for whether the subjects’ response is different from “moderate level (3)” for each item. ^3^ N/A denotes that the question item was not provided in this group of subjects. *** 0.05 level of significance, ** 0.1 level of significance, * non-significant at level of 0.1.

**Table 8:** Description of collected motion data.

Posture Label	Percentage (%)	Posture Label Explanation
BT	14.1	Static bending, minor movement with bending, minor lateral bending, and picking up.
KN	7.3	Kneel on one leg and both legs.
MO	0.8	Postures used when climbing ladders.
SQ	0.3	Squatting.
ST	39.7	Standing with minor movement.
WK	18.0	Walking.
WO	19.7	Overhead work with at least one arm.

**Table 9:** Summary of recognition performance.

Posture	Precision	Recall	F1-Score
BT	0.86	0.86	0.86
KN	0.97	0.97	0.97
MO	0.62	0.57	0.59
SQ	0.89	0.94	0.91
ST	0.90	0.91	0.90
WK	0.86	0.84	0.85
WO	0.94	0.94	0.94
Overall Accuracy	0.90		
Macro F1 Score	0.86		

## Data Availability

The datasets used in this study are available from the corresponding author upon request.

## Appendix A

**Table A1:** Comparison of ergonomics assessment rules.

Assessment Rule	Collected Information	Collection Strategy	Posture Quantification	Evaluation Criteria
Ovako Working Posture Assessment System (OWAS) [27]	Full-body posture	Sampling of observation at a fixed time interval	Quantify posture by a combination of body parts at different approximate positions	Comparing the proportion of classified postures (considering force used) with predefined criteria, deciding any corrections to be undertaken for reducing injury risk.
	Force used			
	The proportion of Posture			
Posture, activity, tools, and handling (PATH) [35]	Full-body posture	Same as OWAS	Same as OWAS	NA
	Force used			
	Work activities			
	The proportion of posture			
Posture Targeting [36]	Full-body Posture	No detailed rules for the collection	Quantify posture by deviation or displacement of body parts from a neutral position.	NA
Task Recording and Analysis on Computer (TRAC) [37]	Full-body Postures	Continuous recording and sampling of observation at a fixed time interval	Quantify posture by both gross posture and rotation or flexion angles of body parts.	NA
	Force			
	Proportion and Distribution of Posture			
Portable Ergonomic Observation (PEO) [38]	Full-body Posture	Continuous recording of postures	Quantify posture by rotation or flexion angles of body parts.	NA
	Force			
	Proportion and Distribution of Posture			
Hand Relative to Body-HARBO [39]	Full-body Posture (mainly standing, walking and sitting) Proportion and Distribution of Posture	Continuous recording of postures	Quantify posture by relative position between hand and body.	NA
Maximum Holding Time (MHT) [28]	Full-body Posture	Continuous recording of static postures	Quantify awkward postures using by relative positions of shoulder and hands.	The MHT thresholds and recommended holding time are provided for varying postures with different level of awkwardness.
	Static holding time of postures			
Rapid Entire Body Assessment Rules (REBA) [40]	Full-body posture	No detailed rules for the collection	Quantify posture by rotation or flexion angles of body parts.	Evaluation score is acquired by cumulating scores regarding different body parts’ positions (considering force used). Injury risk is assessed by comparing to five different risk levels (representing different necessity for actions).
	Force used			
Rapid Upper Limb Assessment Rules (RULA) [41]	Upper limbs’ posture			
	Force used			
Postural Loading on the Upper-Body Assessment (LUBA) [42]	Full-body postures	No detailed rules for the collection	Quantify posture by rotation or flexion angles of body parts.	The perceived posture discomfort score is rated for different posture by experiment, the MHT [28] is also used for assessment.
Chung’s posture workload evaluation system [43]	Full-body posture	No detailed rules for the collection	Quantify body posture by deviation from a neutral position for different joints.	Calculating the whole-body discomfort score by combining discomfort scores from different body parts.
Back-ES [44]	Manual handling posture related to back	A sampling of observation at a fixed time interval	Quantify posture by rotation or flexion angles of body parts.	NA
	Force used			
	Vibration			

**Table A2:** Pseudo-code for real-time MHT Assessment.

Real-Time MHT Assessment
count=0← count of consecutive postures threshold← pre−defiended threshold for each targeted activities ${\mathrm{motion}}_{i}\leftarrow \mathrm{motion}\;\mathrm{data}\;\mathrm{collected}\;\mathrm{in}\;\mathrm{the}\;i_{\mathrm{th}}\;\sec\mathrm{ond}$ PR(motion)←pre−trained posture recognition model $while{\;\mathrm{motion}}_{i}\;not\;\mathrm{empty}:$ # continuously capturing the motion data $\mathrm{pre}=\;\mathrm{PR}\left({\mathrm{motion}}_{i-1}\right)$ # comparing postures detected from two consecutive seconds $\mathrm{cur}=\;\mathrm{PR}\left({\mathrm{motion}}_{i}\right)$ **if** cur **equals** pre: count+ = 1 **if** count > threshold[cur]: # send the warning signal when breaching MHT threshold count = 0 # reset the counter once breaching the MHT threshold **output** “posture cur breaches the MHT threshold” **else**: count = 0 # reset the counter when consecutive postures are different

**Table A3:** Pseudo-code for periodical MHT and posture proportion assessment.

Periodic MHT and Awkward Posture Proportion Assessment
postures ← list of postures (with timestamps) recognized threshold ← pre-defined threshold for each targeted posture result ← result of MSDs risk assessment in the unit time interval pointer = 0 ← pointer for counting consecutive postures *c* ← number of posture classes recognized in the unit working time *n* ← number of postures recognized in the unit working time count ← count the continuous holding time of every posture count [0,1] = 1 ← initialize the first captured posture sub_count ← buffer for saving holding time of specific posture for *i* in range(*n* − 1): if postures[*i* + 1] equals postures[*i*]: count[pointer,1]+ = 1 count[pinter,0] = postures[*i*] else: pointer+ = 1 # the pointer moves to the next consecutive postures when the different posture comes count[poiner,0] = postures[*i* + 1] count[pointer,1] = 1 count[:, column3] = (count[:, column2] + 1)×0.5 # add a third column in count to record time of continuously held posture, the postures recognized under 50% overlap sliding window for *i* in range(*c*): sub_count = count[column1 equals *i* and column2 greater than threshold[*i*]] # all *i* breaching MHT threshold result [*i*, 1] = sub_count.length # total count of posture *i* breaching MHT result [*i*, 2] = sub_count.sum # total duration of posture *i* breaching MHT result [*i*,3] = count[column1 equals *i*].length/(count[column2].sum×60) # frequencies of posture *i* in 1 min result [*i*,4] = count[column1 equals *i*].sum/count[column1].sum # proportion of posture *i* result [*i*,5] = count[column1 equals *i*].max # max holding time of posture *i* return result

**Table A4:** Evaluation survey question design.

System Design Criteria	Metrics		Question Items and Explanation	
			Worker	Manager
Wearable Sensing System	Physical Discomfort Level		Q1: Physical discomfort when wearing the sensors	Q4: Perceived worker’s physical discomfort when wearing the sensors
	Mental Discomfort Level		Q2: Mental discomfort when wearing the sensors	Q5: Perceived worker’s mental discomfort when wearing the sensors
	Inconvenience Level		Q3: Inconvenience caused by the sensors, and sensor locations causing the inconvenience.	N/A
	Intrusiveness Level		Q4: Likelihood of productivity reduction due to the intrusion caused by sensors	Q6: Estimated likelihood of productivity reduction due to sensors’ intrusion
	Durability and Robustness Level		Q5: Durability of the sensors to the construction environment	Q7: Perceived durability of the sensors to the construction environment
	Acceptability of Wearable Sensors		Q6: Likelihood of workers using the sensors	Q8: Perceived likelihood of workers using the sensors
Risk Assessment and Information Delivery System	Content of Assessment	Usefulness of Immediate Intervention	Q7: Usefulness of providing real-time intervention for individual workers	Q12: Perceived usefulness of providing real-time intervention for individual workers
		Usefulness of a Periodical Assessment	Q8: Usefulness of providing periodical assessment for individual workers (every 30 min)	Q14: Usefulness of providing daily time profile of awkward postures for refining break schedule Q15: Usefulness of identifying recurring awkward postures for trades and trades with high awkward posture exposure
		Trust in Proposed Assessment	Q9: Level of confidence that the evaluation can give useful information for individual workers	Q16: Level of confidence the evaluation can give useful information for jobsite
Risk Assessment and Information Delivery System	Mobile App-based Assessment Information Delivery for Individuals	Incidence of the recurring awkward posture of individuals	Q10: Usefulness of providing incidence of recurring awkward postures time profile of awkward postures immediate intervention for individual workers via the App	Q13: Perceived usefulness of App-based assessment information delivery
		Daily time profile of awkward postures		
		Need for taking immediate intervention		
	Online Dashboard-based Assessment Information Delivery for Jobsite	Daily time profile of awkward postures	N/A	Q17: Usefulness of providing: daily time profile of awkward postures on jobsite types of awkward postures by trade injury risk levels of trade recommended speed of taking intervention for trades
		Incidence of recurring awkward postures by trades		
		Injury risk level by trade		
		Recommended speed of taking intervention by trade		
Risk Assessment and Information Delivery System	Frequency of Information Delivery	Real-time	Q11: Perceived effectiveness of delivering the individual assessment information at the frequency of: Real-time alarm for breaching MHT Assessment summary in every 30 min Daily assessment summary	Q18: Perceived effectiveness of Delivering the individual assessment information at the frequency of: Real-time alarm for breaching MHT Assessment summary in every 30 min Daily assessment summary Delivering jobsite assessment information at the frequency of: Daily jobsite summary
		Every 30 min		
		Daily assessment		
Holistic Evaluation	Potential System Usefulness		Q12: Perceived usefulness of the developed DSS system by workers	Q1: Perceived usefulness of the developed DSS system by managers
	System Applicability		N/A	Q2: Perceived applicability of the developed DSS system on jobsite by the managers
	Likelihood of Adoption		Q13: Likelihood of using the developed DSS in workers’ daily work	Q13: Likelihood of using the developed DSS in the managers’ daily work
